# Supplementary material for: Microsoft Bing outperforms five other generative artificial intelligence chatbots in the Antwerp University multiple choice medical license exam
Source: PLOS Digit Health. 2024 Feb 14;3(2):e0000349. doi: 10.1371/journal.pdig.0000349 (PMC10866461; doi:10.1371/journal.pdig.0000349)
Supplement: S1 Table — (DOCX) [file pdig.0000349.s001.docx]

## S1 Table. Example of generative chat bot answers to an exam question.

| Question | Bing | ChatGPT | Bard | GPT-4 | Claude Instant | Claude+ |
| --- | --- | --- | --- | --- | --- | --- |
| **The brain can obtain energy from:**   1. glucose and fatty acids 2. **glucose and ketone bodies** 3. glucose and proteins 4. proteins and fats | **The brain can obtain energy from glucose and ketone bodies.** Glucose is produced when carbohydrates break down in your body and is then used as energy^1^. When glucose levels are limited, ketone bodies generated in the liver can also become important energy substrates for the brain^2^. So, the correct answer would be option b) glucose and ketone bodies.  ^1^healthfully.com  ^2^nature.com | The correct answer is option (a) glucose and fatty acids.  *Glucose and fatty acids are the primary sources of energy for the brain.* Glucose can be obtained from the breakdown of carbohydrates in the diet or from the liver and muscles, which store glucose in the form of glycogen. Fatty acids can be obtained from the breakdown of fats in the diet or from adipose tissue, which stores fat in the form of triglycerides.  While the brain can also use ketone bodies as an alternative source of energy during periods of fasting or prolonged exercise, this is not a primary source of energy for the brain. Similarly, while proteins can be broken down into glucose through a process called gluconeogenesis, this is not a preferred source of energy for the brain as it can lead to the depletion of muscle mass. | **The answer is a) glucose and fatty acids.**  The brain is the most energy-demanding organ in the body, and it relies on glucose as its main source of energy. However, the brain can also use fatty acids for energy, especially during times of fasting or starvation. Ketones are a type of fuel that the body can produce from fatty acids, and they can also be used by the brain for energy. However, the brain cannot use proteins for energy.  Here are some additional details about how the brain obtains energy: … | **The brain can obtain energy from: b) glucose and ketone bodies** | The answer is a) glucose and fatty acids. Here are the details:  The brain requires a constant source of energy to function properly. It obtains this energy primarily from glucose and fatty acids.   Glucose: Glucose is the brain's primary fuel source. Glucose enters the brain from the bloodstream and is broken down by the cell to provide energy. Glucose is most readily available as an energy source.   Fatty acids: Fatty acids can also provide energy for the brain, though to a lesser extent than glucose. Fatty acids are broken down in the liver into ketone bodies which can then enter the brain and provide energy. However, the brain prefers to use glucose.   Ketone bodies: Ketone bodies, like acetoacetate and beta-hydroxybutyrate, can provide some energy to the brain. However, the brain obtains most of its energy from glucose, not ketone bodies.   Proteins: The brain cannot efficiently utilize proteins as an energy source. Proteins are primarily used for building and maintaining brain cells, not for energy.  So in summary, the brain's main sources of energy are glucose and fatty acids, broken down into ketone bodies to a lesser extent. Proteins and fats alone cannot provide significant energy for the brain.  Hope this explanation helps! Let me know if you have any other questions. | The correct answer is:  a) glucose and fatty acids  The brain primarily relies on glucose and fatty acids for energy. Ketone bodies can also be used as an alternative energy source, but proteins are not a major source of energy for the brain. So options b and c are incorrect. And while the body can use proteins and fats for energy in general, the brain preferentially uses glucose and fatty acids. So option d is also incorrect.  The brain is very dependent on glucose, but it can adapt to use ketone bodies during times of fasting or low-carb diets. But proteins are more structural components and precursors for neurotransmitters - they are not a primary fuel for the brain. Fatty acids are also readily taken up and used for energy by the brain.  So in summary, the brain relies most on glucose and fatty acids for its energy needs. Options a is the correct choice. |

**Bold:** correct statement
