# Supplementary material for: Microsoft Bing outperforms five other generative artificial intelligence chatbots in the Antwerp University multiple choice medical license exam
Source: PLOS Digit Health. 2024 Feb 14;3(2):e0000349. doi: 10.1371/journal.pdig.0000349 (PMC10866461; doi:10.1371/journal.pdig.0000349)
Supplement: S2 Data — (DOCX) [file pdig.0000349.s003.docx]

S2 Data: Variable overview

| **Variable** | **Data type** | **Explanation** |
| --- | --- | --- |
| Question | Numeric | Number of the question |
| Solution | Categoric: a, b, c or d | Correct answer |
| Negative question | Dichotomous: yes/no | Yes: this question was negatively formulated |
| Diffculty | Categoric: easy, moderate or difficult |  |
| SecondBestAnswer | Categoric: No, a, b, c or d | No: there was no second best answer for this question |
| FatalAnswer | Categoric: No, a, b, c or d | No: there was no fatal answer for this question |
| ClinicalVignette | Dichotomous: yes/no | Yes: this questions describes a clinical situation  No: theory question |
| *Bot*Strict | Categoric: a, b, c, d, Unclear,  NoAnswer,  SeveralAnswersWithoutChoice,  Or RefusalToAnswer | Answer of the bot |
| *Bot*StudentScore | Categoric: -1, 0, 0.33333, 1 | See paper |
| *Bot*Score | Categoric: 0 or 1 | See paper |
| *Bot*ScoreAdapted | Combination of *Bot*Score and *Bot*Strict | Made to ease interpretation |
| *Bot*Hallucination1 | Categoric: No, Untruthful or ReasoningError | Scored by author VV. See methods why we have combined Untruthful and ReasoningError in the report. |
| *Bot*Hallucination2 | Categoric: No, Untruthful or ReasoningError | Scored by author DM. See methods why we have combined Untruthful and ReasoningError in the report. |
| *Bot*HalluciationFinal | Categoric: No, Untruthful or ReasoningError | Scored by author SM. See methods. |
