# Supplementary material for: Microsoft Bing outperforms five other generative artificial intelligence chatbots in the Antwerp University multiple choice medical license exam
Source: PLOS Digit Health. 2024 Feb 14;3(2):e0000349. doi: 10.1371/journal.pdig.0000349 (PMC10866461; doi:10.1371/journal.pdig.0000349)
Supplement: Attachment — Submitted filename: Rebuttal Letter AI chat bots.docx [file pdig.0000349.s004.docx]

Dear editor, dear reviewers,

Thank you for your suggestions and for allowing us a revision. We believe we addressed all the remaining issues and are willing to make additional changes if necessary. Please find our response to each raised comment in the table below. In addition, we have correct some very minor typesetting, grammar and spelling errors. These changes are visible in the tracked changes version of the revised manuscript. The reference list has been revised entirely.

Sincerely,

The authors

| **Reviewer 1** | |
| --- | --- |
| **Comment** | **Response** |
| The ensemble bot's introduction in line 133 requires further elaboration. The citation provided broadly addresses ensemble methods. For a clearer comprehension of the study's scope, it would be beneficial to offer an overview of this ensemble bot's specifics, including how to use it, and its primary functionalities. Further, its role and comparative significance in relation to other bots merit some discussion. | We have added details concerning ensemble bots and their usefulness to the methods section including reference 22 (textbook concerning ensemble learning). The ensemble bot we used was just the most common value (mode) of the answers of all six bots, we have rephrased this description to improve readability.  We have also added a statement to the discussion concerning this ensemble bot. |
| In line 265, the statement alluding to the creators' intentions to restrict chatbots from being utilized as medical doctors could use some evidential support. A citation or reference validating this assertion would enhance the credibility of the claim. Additionally, considering the advancements in AI medical applications, such as Google's MedPalm, it might be worthwhile to delve into the potential aspirations of some companies to position their chatbots as medical consultants. | We have completely revised this paragraph with a more profound discussion on current literature including your useful suggestion concerning MedPalm. |
| In line 196 I noticed an inconsistency in the reporting of p-values within the manuscript. Specifically, the use of a percentage sign after the p-value in the line "P=0.06%" is atypical. Generally, p-values are reported as decimals, such as "P=0.0006" or "P<0.01". I recommend standardizing the presentation of p-values throughout the manuscript for clarity and consistency. | This was a flaw, the “%” has been removed and consistency of the presentation of all p-values has been checked. |
| The statement in the conclusion, "Bots should improve their algorithm if they do not want to be used as a medical," could be further refined for clarity, perhaps by rephrasing it to "used in a medical capacity." | This was a typesetting error, the last part of this sentence was probably accidently deleted. We have corrected this sentence: “Companies should improve their bot’s algorithm if they do not want them to be used as tool for medical advice.” |
| The suggestion that Bing can serve as a tool for identifying weaker questions is intriguing. Elaborating on the mechanics of this process and its efficiency relative to other methods would further solidify this proposition. | We have added a reflection to the discussion section. We were unable to find other authors that gave the same suggestion. |
| **Reviewer 2** | |
| Improve your references to the literature, have a literature review section. Inform your reader what other work did before and what it is your work is adding to the literature | We have adopted this suggestion and added many recent citations. See comments of reviewer 1 above for more details. |
| Organization: what the difference is between (overall) outcome and (detailed) results sections may be confusing to the reader | We have revised the subtitles in the results section. |
